# Projection-Angle-Sensor-Assisted X-ray Computed Tomography for Cylindrical Lithium-Ion Batteries

**DOI:** 10.3390/s24041102

**Published:** 2024-02-08

**Authors:** Jiawei Dong, Lingling Ju, Quanyuan Jiang, Guangchao Geng

**Affiliations:** 1 College of Electrical Engineering, Zhejiang University, Hangzhou 310027, China; 2 Polytechnic Institute, Zhejiang University, Hangzhou 310015, China; 3 International Research Center for Advanced Electrical Engineering, Zhejiang University, Haining 314499, China

**Keywords:** XCT, lithium-ion batteries, error analysis, angle sensor, 3D reconstruction

## Abstract

X-ray computed tomography (XCT) has become a powerful technique for studying lithium-ion batteries, allowing non-destructive 3D imaging across multiple spatial scales. Image quality is particularly important for observing the internal structure of lithium-ion batteries. During multiple rotations, the existence of cumulative errors and random errors in the rotary table leads to errors in the projection angle, affecting the imaging quality of XCT. The accuracy of the projection angle is an important factor that directly affects imaging. However, the impact of the projection angle on XCT reconstruction imaging is difficult to quantify. Therefore, the required precision of the projection angle sensor cannot be determined explicitly. In this research, we selected a common 18650 cylindrical lithium-ion battery for experiments. By setting up an XCT scanning platform and installing an angle sensor to calibrate the projection angle, we proceeded with image reconstruction after introducing various angle errors. When comparing the results, we found that projection angle errors lead to the appearance of noise and many stripe artifacts in the image. This is particularly noticeable in the form of many irregular artifacts in the image background. The overall variation and residual projection error in detection indicators can effectively reflect the trend in image quality. This research analyzed the impact of projection angle errors on imaging and improved the quality of XCT imaging by installing angle sensors on a rotary table.

## 1. Introduction

X-ray computed tomography (XCT) was originally used in the medical field. In recent years, it has gradually been applied in the industrial domain for detecting material defects and measuring dimensions [1,2]. This technology has been utilized in various areas such as materials, measurement and manufacturing, engineering, food, biology, geology, and paleobiology [3]. As it allows for non-destructive 3D imaging of complex systems across various spatial scales to obtain key information about the battery structure and dynamic processes, such as the lithiation kinetics and degradation that occur during battery operation, XCT has become a formidable tool in the study of lithium-ion batteries [4]. At the cell level, an inspection of the internal structure, including the thickness of the anodes and cathodes, the density of accumulation, the arrangement, and the presence of internal gases [5], allows for the investigation of macroscopic defects introduced during battery design and manufacture [6]. Furthermore, this provides insight into battery operation and degradation [7], as well as into structural damage inside the battery in the event of thermal runaway [8]. At the microstructural level, X-ray tomography is commonly used for quantitative analyses of electrode structures to determine parameters such as the particle size, tortuosity [9], and volume fraction [10]. In addition, X-ray tomography can also be used to analyze dynamic processes such as lithium kinetics and degradation [11].

Research on lithium-ion batteries requires high-precision XCT to observe internal structures and often needs size measurements. Therefore, the quality of XCT images, which includes factors such as the resolution, distortion, and artifacts, is crucial for studying lithium-ion batteries. However, as many factors can affect the quality of the images, obtaining high-quality XCT images is not easy [12]. These factors can be divided into five groups of parameters influencing the operation: system, workpiece, data processing, environment, and operator. System-related errors can further be divided into the X-ray source, X-ray detector, and positioning system [13]. Beam hardening can result in cupping artefacts and streaking artefacts [14]. Beam fluctuations can cause extensive ring artifacts. If the pixels of the X-ray detector have defects, or if the scintillator is locally dirty or defective, this can result in ring artifacts appearing in the image [4]. Errors in the rotation center can cause the image to appear blurred [15]. In the positioning system, the positioning accuracy of the rotary table is an important factor affecting the quality of XCT images. This is because the projection path of the X-ray is determined by the projection angle. If the projection angle changes, the penetration path of each layer will change, resulting in a change in the qualities of the projected image. If the projection angle changes, the corresponding data in the reconstruction process will change, causing distortion and artifacts in the reconstructed image. Another serious problem is that if there is an angular error, this error will accumulate in all the projection images, and even if the angular error of each projection image is very small, their cumulative effect may cause large distortions and artifacts in the reconstructed three-dimensional image. Such distortions and artifacts can not only affect the quality of the image but may also affect the analysis and understanding of the internal structure of the object.

A lot of research has been conducted on the angle of projection, including studies on limited projection angles [16] and incomplete projection [17]. However, few research works have focused on the impact of projection angle errors on image quality. An algorithm to correct the projection errors of synchrotron X-ray computed tomography yielded good results [18], but there has not been any related research on the sensors involved in projection. In terms of the accuracy of projection angles, people often purchase rotary tables with a higher precision, then pay no further attention to the errors of the rotary table. As XCT requires rotating to specified angles hundreds or even thousands of times, unavoidable cumulative errors and some incidental errors exist. As a result, the actual projection errors are often larger than the positioning precision of the rotary table.

In tomographic 3D reconstruction, the set rotational angle interval of the rotary table is typically multiplied by the number of rotations to determine the projection angle. However, in practical use, the rotary table has rotational errors, and since there is no angle monitoring device, we cannot know the actual degree of rotation of the rotary table. There is no need to purchase a high-precision rotary table. By installing suitable angle sensors on the rotary table, we can obtain the real-time rotation data of the rotary table and thus adjust the original projection angle, greatly improving the accuracy of the projection angle. Due to the widespread application of 18650 lithium-ion batteries, many researchers have chosen them as their subject of study [19,20]. However, the projections in all directions of cylindrical lithium-ion batteries are similar, making it difficult to spot errors in the projection angle. Therefore, cylindrical lithium-ion batteries are selected for experiments. For the study of XCT imaging quality, people often establish virtual projection models on a computer for simulation analysis. However, simulations cannot adequately reflect the actual XCT scanning situation for systems with complicated internal structures like lithium-ion batteries. Therefore, experiments use real projection data from cylindrical lithium-ion batteries. In experiments, the total variable [18,21,22] and residual projection errors [23] are used as indexes of the reconstructive image quality.

This paper uses an angle sensor to correct the projection angle and analyzes the impact of projection angle errors on cylindrical lithium-ion battery XCT imaging. The Section 2 discusses the principles and reconstruction algorithm of cylindrical lithium-ion batteries. The Section 3 introduces the principles of the rotary table and projection angle sensor in XCT imaging. The Section 4 introduces the experimental process, including the construction of the experiment platform, data processing, image reconstruction, and analysis. The Section 5 provides the conclusion.

## 2. X-ray CT for Cylindrical Lithium-Ion Batteries

### 2.1. Basic Principle of X-ray CT

**Figure 1:**
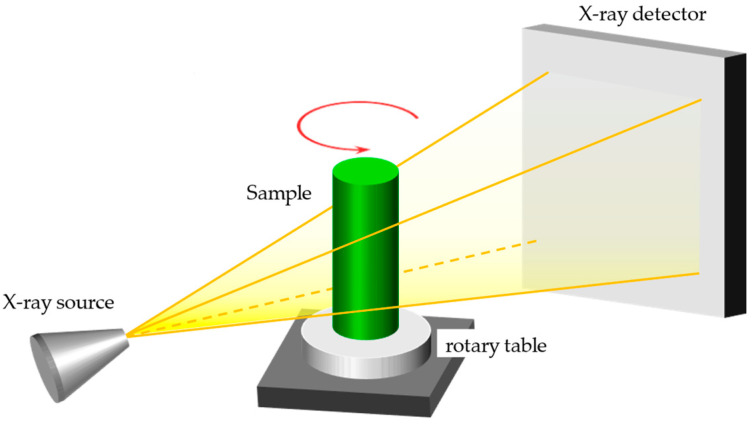
Cone-beam computed tomography scanners.

A typical XCT system consists of an X-ray source, a mechanical scanning motion system, a radiation detector system, and a computer system to analyze the received data. Typical cone-beam computed tomography (CBCT) is shown in Figure 1. During operation, the X-ray tube emits an X-ray beam. As this beam penetrates an object, the interactions between the X-rays and the material decrease the intensity of the X-rays in the direction of incidence. The X-ray detector records the degree of attenuation of the X-ray beam as it passes through the object. The intensity value of each pixel in the detector is a function of the attenuation coefficient (*µ* (*s*)) and the path of the X-rays, and the variation in attenuation intensity adheres to the Beer–Lambert law [24]:

(1) $$p_{L}=ln\left(I_{0}/I\right)=\int_{0}^{L}\mu \left(s\right)ds.$$

where *I*_0_ is the incident X-ray intensity and *I* is the intensity after interaction with the matter. The length of the path through the matter is *L*, and the attenuation coefficient of the matter is *µ* (*s*).

Through the rotation of the rotary table, we can obtain the projection of the object at different angles and positions, and thereby the attenuation coefficients *µ* (*s*) of each point inside the object can be reconstructed. *µ* (*s*) is approximately proportional to the density of the object. So, we can use *µ* (*s*) to distinguish different parts inside an object, achieving the goal of detecting the internal structure of the object.

Based on the shape of the X-ray beam, industrial CT scanning is primarily divided into two categories: fan beams and cone beam X-rays. Compared to fan beam X-rays, cone-beam CT scanners can acquire three-dimensional volume data in a single rotation, which leads to a higher utilization rate of the rays and a faster scanning speed. Therefore, the mainstream commercial CT machines in the industrial field are currently CBCT scanners [25].

### 2.2. Three-Dimensional Reconstruction Algorithm

Currently, analytical algorithms and iterative algorithms are the two main types of algorithms used for three-dimensional cone beam CT image reconstruction, where analytical algorithms can be further divided into approximate and exact algorithms. Usually, solving integral equations requires less memory space and is faster than solving systems of equations, so analytical algorithms can complete image reconstruction more quickly and occupy less storage space. Iterative algorithms provide more accurate results and better noise suppression, but they are slower and use more memory. In the actual application process of industrial non-destructive testing, it is usually necessary to obtain reconstruction results at a fast speed; hence, analytic algorithms have become the mainstream algorithms used in practice in cone beam CT and are widely used in various fields.

The most classic algorithm in approximate algorithms is the Feldkamp–Davis–Kress (FDK) [26] reconstruction algorithm proposed by Feldkamp, Davis, and Kress in 1984. This algorithm is a three-dimensional extension of the two-dimensional fan-beam filter backprojection algorithm. Its main steps can be summarized as the weighting of projection data, filtering, and backprojection. Although it is an approximate solution from a mathematical point of view, due to its simple implementation and high computational efficiency, it quickly became the most popular algorithm in CT industrial applications. The FDK algorithm can approximate the reconstruction of the image well when the cone angle is small, and at the center plane, the FDK algorithm formula can be converted into the fan-beam filter backprojection reconstruction formula; that is to say, it is completely equivalent to the two-dimensional fan beam at the center plane and is an exact reconstruction. As the reconstruction plane moves further away from the center plane, the reconstruction error increases. Appropriately increasing the amount of projection data can effectively improve the quality of the reconstructed image.

### 2.3. XCT Detection of Cylindrical Lithium-Ion Batteries

There are several lithium-ion cell architectures available on the market, such as pouch cells, prismatic cells, and cylindrical cells. Among them, cylindrical lithium-ion cells, especially 18650 batteries, are favored due to their high energy density and large storage capacity. They are widely used in various devices, including power tools, laptops, electric bicycles, and electric vehicles [19,20].

**Figure 2:**
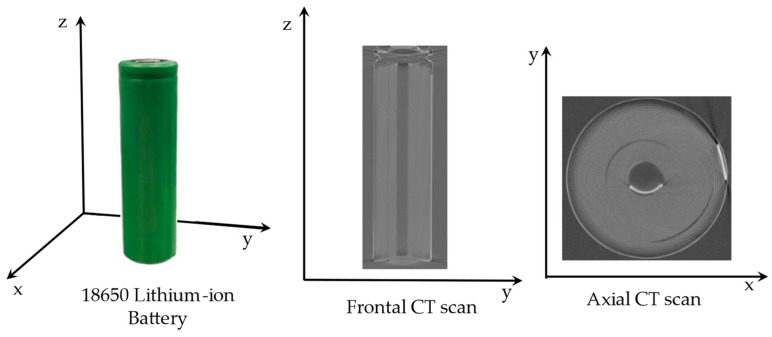
XCT scan images of a cylindrical lithium-ion battery.

The structure of cylindrical lithium-ion batteries mainly consists of two components. The first is the jelly roll, which is created by winding a composite material made up of a cathode, an anode, and two separators [27]. The second is the cell housing, constructed from a can and a cap. The larger the atomic number of the general element, the greater the X-ray attenuation and gray value [28]. The anode current collector in lithium-ion batteries is typically made from copper foil, with nickel often used for the negative electrode plate. The primary materials for the cathode current collector are usually nickel and aluminum, while the anode material is typically carbon. Elements with higher atomic numbers, such as those in the anode current collector, have higher X-ray attenuation and grayscale values, making them appear white in CT images. In contrast, elements with lower atomic numbers, like those in the anode material, present lower grayscale values and appear black in CT images [6]. XCT scanning images of a cylindrical lithium-ion battery are shown in Figure 2.

Several studies have investigated structural changes in cylindrical lithium-ion batteries due to aging or defects. For example, Waldmann et al. [29] explored jelly roll deformation in commercial, pin-less 18650 batteries under varying C-rate cycles. Yi Wu [6] and colleagues used computed tomography scanning to study defects and structural deformations caused during the manufacturing of lithium-ion batteries. In addition, Pavel Blazek [30] has conducted research on the uneven axial and radial expansion of commercial 18650 lithium-ion batteries during the cycling aging process. During the XCT scanning of cylindrical lithium-ion batteries, due to the symmetry between their internal and external circular structures, the projected images at various angles are quite similar. It is not easy to observe if there are any errors in the projection angle, which can affect the quality of the images.

## 3. CT Imaging Quality Improvements Using Projection Angle Sensors

### 3.1. Projection Angles in CT Imaging

The geometric shape of an industrial CT system is defined by the relative position and direction of three components: the X-ray source, the rotary table, and the X-ray detector [31]. The rotation accuracy of the rotary table directly affects the accuracy of the XCT projection angle.

There are many different types of rotary tables available on the market, where either direct drive or various types of mechanical gear transmissions are employed [32]. The structure of the rotary tables that are driven by direct drive motors is simpler and avoids wear and tear caused by transmission, resulting in a better dynamic performance and a higher positioning accuracy. However, the cost is relatively high. The rotary tables which use mechanical gear transmission are driven by stepper motors or servo motors and are rotated through gear reduction mechanisms like gears and worms to drive the actuator. The rotation fluctuation, backlash, and rigidity directly influence the performance of the motion rotary table [33]. This type of rotary table has a high positioning accuracy and a low cost, which makes it widely used.

The resolution, positioning accuracy, and repetitive positioning accuracy are three important indicators of a rotary table. Resolution refers to the smallest motion increment that the system can generate, which directly determines how finely the rotary table can operate at different angles. The positioning accuracy indicates the maximum deviation between the actual position and the target position after the rotary table moves from a specified initial position to a specified target position, reflecting the ability of the rotary table to accurately arrive at the set point. The repetitive positioning accuracy refers to the statistical characteristics of the position error when the rotary table terminal returns to the same position multiple times under the same operating conditions, reflecting the stability and reliability of the rotary table. Generally, repeatability is higher than the positioning accuracy.

### 3.2. Projection Angle Sensors

Angle sensors can be classified as capacitive angle sensors, inductive angle sensors, angle sensors based on the Hall effect and magnetoresistive effects, and optical angle sensors. Among these, optical sensors are widely used due to their high precision and excellent resolution coupled with a strong resistance to magnetic interference [34].

**Figure 3:**
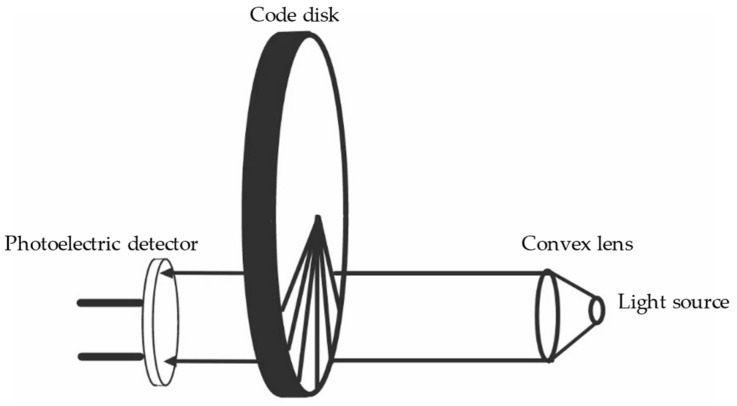
The principle of optical angle encoders.

Most optical angle sensors operate based on encoder technology. They function by converting mechanical measures into pulses or digital quantities through photoelectric conversion. A photoelectric encoder, which is shown in Figure 3, is composed of a light source, a photoelectric detector, and a code disk [35,36]. The code disk has equally spaced transparent and non-transparent slits. The rotational axis moves in synchrony and speed with the code disk, and the detector is made up of electronic components like light-emitting diodes. The pulse signals passing through the slit are output by the detector and the current angle information of the rotating shaft can be reflected by calculating changes in the pulses output by the photoelectric encoder every second.

There are two types of photoelectric encoders: one is an incremental photoelectric encoder, and the other is an absolute photoelectric encoder [37,38]. Incremental encoders generate a series of pulses as the axle rotates the coded disk, then, depending on the direction of rotation, a counter is used to increase or decrease the counts of these pulses in order to represent the angle displacement. Any position on the disk of an absolute encoder has a fixed digital code corresponding to that position, and it directly outputs digital readings by interpreting the pattern information on the disk. Absolute encoders are not affected by power outages and have a good anti-interference performance. However, under the same resolution conditions, the structure of incremental encoders is simpler and their price is also cheaper than that of absolute encoders; thus, incremental encoders have their own advantages [37,39]. The working mode of industrial CBCT typically involves conducting an XCT scan with a rotary table after each rotation of a certain angular interval to obtain projection data until the specified scan angle is completed. Therefore, we only need to know the angle relative to the initial angle, and there is no need to consider the loss of absolute angle during a power reset. As such, incremental encoders can effectively satisfy the requirements of industrial CT use. The development of current incremental encoders is quite advanced and they can achieve a high accuracy. The resolution of most high-precision encoders can reach over 10,000 pulses per revolution (PPR), which can meet the requirements for inspections of the internal structure of batteries.

One of the key factors to consider when choosing a projection angle sensor is its resolution. To ensure the accuracy of measurements, the resolution of the projection angle sensor should be higher than the rotary table. Moreover, in order to maintain a high measurement accuracy, it is also very important to select sensors with a high output, precision, and stability. It is critical to pay special attention to the installation and fixation of the projection angle sensor. Using a stable fixing structure can ensure the sensor’s stability during the measurement process, preventing measurement errors due to vibrations or other factors.

## 4. Laboratory Experiment

### 4.1. Test Platform Setup

**Figure 4:**
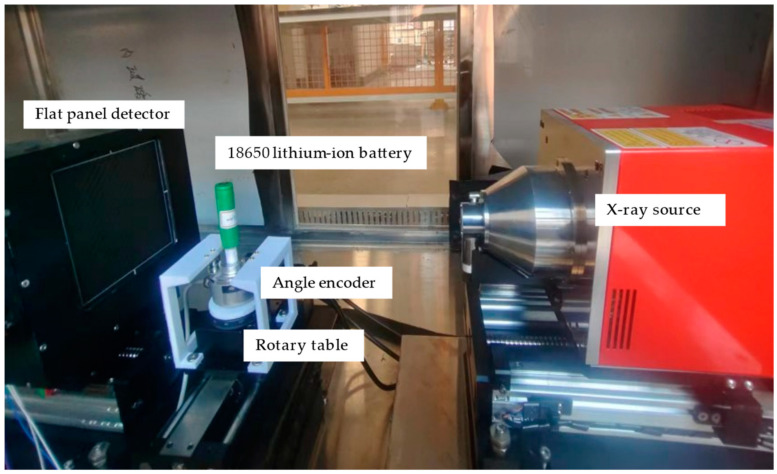
X-ray CT platform.

An X-ray CT platform was designed and built to test the impact of angular projection errors on the X-ray CT of lithium-ion batteries. A schematic diagram of the X-ray CT platform is shown in Figure 4. In order to test the impact of projection angle errors on the X-ray CT of lithium-ion batteries, a high-precision angle sensor was installed on the X-ray CT device’s rotary table to correct angle deviations. The angle sensor uses an optical encoder and was fixed onto the rotary table using 3D-printed components. 

For X-ray CT imaging, we used cone beam CT imaging in this study. Our X-ray CT system consists of an X-ray source, a flat panel detector, a rotary table, and an encoder. The X-ray source is a 90 KV micro-focus X-ray source (Unicomp Technology, Hamamatsu, Japan), and the flat panel detector is an NDT 0505 J low-noise flat panel sensor (iRay, Shanghai, China). The total pixel area is 13 cm × 13 cm, the total pixel matrix is 1536 × 1536, the pixel size is 85 microns, and the maximum frame rate is 40 fps. The rotary table is an HGC3 rotary table (Yunke, Xiajin County, China), which is controlled by a stepper motor with a repeat positioning accuracy of 0.01°. The encoder is a K50-T6C1024B15 incremental photoelectric encoder (Hengxiang, Shanghai, China), with a resolution of 40,000 PPR. The battery chosen for the experiment is an 18650 lithium-ion battery.

The X-ray imaging experiment was conducted under the conditions of 80 kV, 80 μA, and 6.4 W. During sampling, the images were collected every 5 s after the rotary table rotated 0.5°, and a total of 720 images were collected. The distance between the X-ray source and the rotary table was 20.0 cm, and the distance between the flat panel detector and the rotary table was 18.5 cm.

### 4.2. Reconstruction Quality Metric

Artifacts are a significant factor affecting the quality of CT imaging. These anomalies that appear on images do not represent actual structures but are generated by the hardware of the device or software during the imaging process. Recently, total variation (*TV*) has been used as a measure of reconstruction quality in studies of algorithms for reducing CT artifacts. Emil Y Sidky and Xiaochuan Pan [22] proposed an algorithm called adaptive-steepest-descent POCS (ASD-POCS), which minimizes the *TV* of images to reduce artifacts when the angle range is limited or the angle sampling rate is low. When Chang-Chieh Cheng et al. [18] researched the correction of the rotation center and projection angle in synchrotron X-ray computer tomography, they used the gradient descent algorithm to reduce the *TV* value to correct the error in the rotation center and projection angle. Their algorithms have achieved very good results. The *TV* is defined as in Equation (2) [18]:

(2) $$TV\left(I\right)=\sum_{y=1}^{m}\sum_{x=1}^{n}\left|I^{\prime }\left(x,y\right)\right|,$$

where

(3) $$I^{\prime }\left(x,y\right)={\left[I_{x},I_{y}\right]}^{T}={\left[\frac{I\left(x+1,y\right)-I\left(x-1,y\right)}{2},\frac{I\left(x,y+1\right)-I\left(x,y-1\right)}{2}\right]}^{T},$$

and

(4) $$\left|I^{\prime }\left(x,y\right)\right|=\sqrt{I_{x}^{2}+I_{y}^{2}}.$$

The *m* and *n* represent the total number of pixels in the image and *I (x, y)* represents the intensity of pixel (*x*, *y*) in the image. As per the definition, the *TV* is the sum of squares of differences between all pixels and their neighboring pixels. $I^{\prime }$ is an operator that enhances high-frequency signals, such as edge detection. Projection angle errors can cause artifacts of arcs and lines, which increase the *TV*; therefore, the *TV* is suitable for measuring the quality of fault reconstruction. In this study, we chose the *TV* as an indicator of image reconstruction.

**Figure 5:**
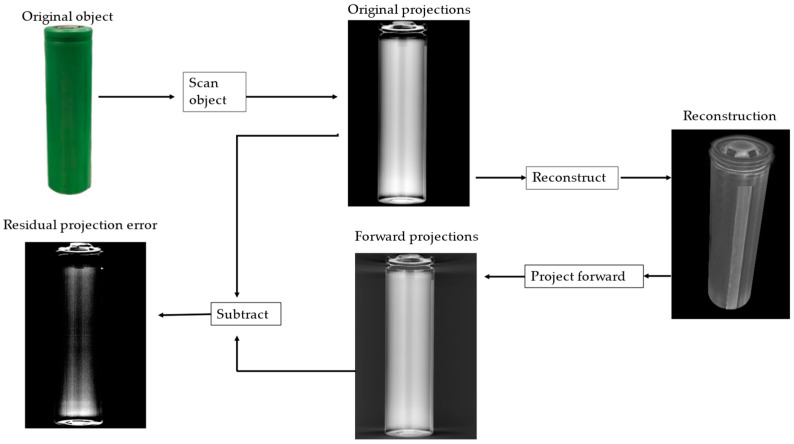
Overview of the computation of the residual projection error.

In addition, we used the residual projection error to analyze the accuracy of the generated images. Figure 5 provides an overview of the calculation of residual projection. In order to calculate the residual projection error, we forward projected the reconstructed image to obtain the forward projection, then subtracted the original projection from the forward projection to obtain the residual projection and used the absolute difference between the two projections to analyze the accuracy of the reconstruction.

The calculation formula for absolute difference (*AD*) is:

(5) $$AD=\sum_{y=1}^{m}\sum_{x=1}^{n}\left|I_{a}(x,y)-I_{b}(x,y)\right|,$$

where *I_a_* (*x*, *y*) represents the intensity of the pixel (*x*, *y*) in the image of original projections and *I_b_* (*x*, *y*) represents the intensity of the pixel (*x*, *y*) in the image of forward projections. The smaller the value of the absolute difference, the lower the reconstruction error.

### 4.3. Image Processing

In this study, we used the open-source software package Tomographic Iterative GPU-based Reconstruction Toolbox [40] version 2.2 in MATLAB to perform a 3D reconstructions of battery projections using the FDK algorithm. The process of XCT imaging may be affected by the electronic noise of the detector, the electromagnetic noise, and other interferences. In order to reduce the influence of background noise and obtain an accurate projection intensity, we used the open-source image processing package Fiji [41] to process the images. First, we performed XCT scans without inserting the battery to obtain the original background image, which captured the background noise generated by the device itself. Then, using the image processing function of the Fiji software package, we subtracted the original background image from the acquired projection image of the battery, thereby reducing the influence of background noise. Ring artifacts can be caused by differences in the detector unit sensitivity and the instability of the ray intensity. The spatial domain manifestation of ring artifacts is concentric bright or dark areas, while in the frequency domain, they appear as certain discontinuous or vertical frequency components. Therefore, by transforming the 3D image into a sine image, using the FFT to transform the image from the spatial domain to the frequency domain, and then removing the vertical frequency components, we can eliminate ring artifacts. Afterwards, we used MATLAB R2020b software to perform 3D reconstruction of battery projections again.

During the projection reconstruction, the return value from the angle encoder was used to correct the projection angle, achieving an accurate projection angle. Then, a certain degree of angle error was added to this projection angle before reconstruction, and the change in image quality was observed. Assuming that the projection angle deviation follows a normal distribution with a mean of 0, the standard deviation σ was set at 0.01°, 0.03°, 0.05°, 0.1°, 0.2°, and 0.3°.

### 4.4. CT Imaging with Different Projection Angle Errors

Table 1 shows the *TV* and *AD* values at different angle errors σ, which, respectively, represent the quantity of image artifacts and the degree of distortion. It can be observed that as the angle error σ increases, the *TV* value gradually increases. This rise in the *TV* value indicates an increase in image noise and artifacts, leading to a gradual deterioration in image quality. Notably, when the angle error σ is small, the increase in the *TV* value is relatively modest. However, when the error angle σ exceeds 0.1°, the *TV* value increases by 3.4%, and the growth in the *TV* value starts to become notable. Even further, when the error angle σ reaches 0.3°, the *TV* value has already grown by 11.6% compared to its initial state. The trend in AD values is similar to the trend in *TV* values, gradually increasing with the increase in angle error σ, which indicates that the accuracy of the image gradually decreases and becomes distorted. When the error angle σ exceeds 0.1°, the *TV* value increases by 5.2%, and when the error angle σ reaches 0.3°, the AD value has increased by 12.0% from its initial state.

**Figure 6:**
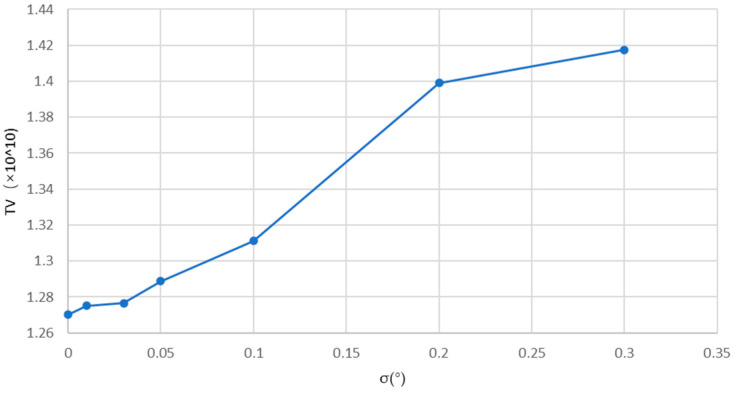
Change curve of *TV* value.

Figure 6 presents the trend in *TV* values with the change in error angle σ. As can be seen, the *TV* value basically increases linearly with the increase in σ, which to some extent demonstrates the excellent performance of the *TV* value as an indicator to reflect angle errors.

**Figure 7:**
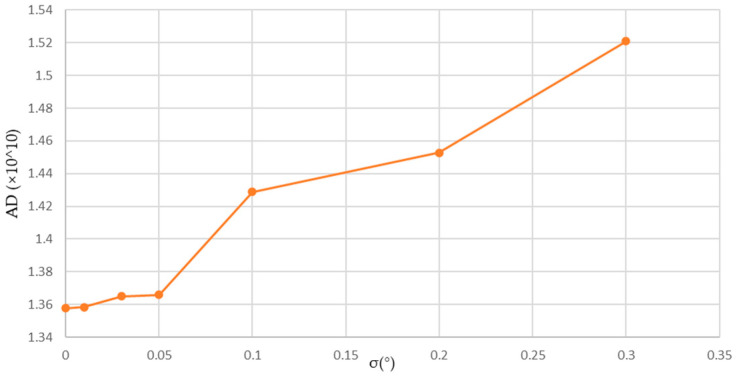
Change curve of absolute difference.

Figure 7 shows the curve of the residual projection error as the error σ changes. The trend is similar to Figure 6, with the change not being too noticeable when σ is less than 0.05°. However, it becomes more significant when σ is greater than 0.05°.

**Figure 8:**
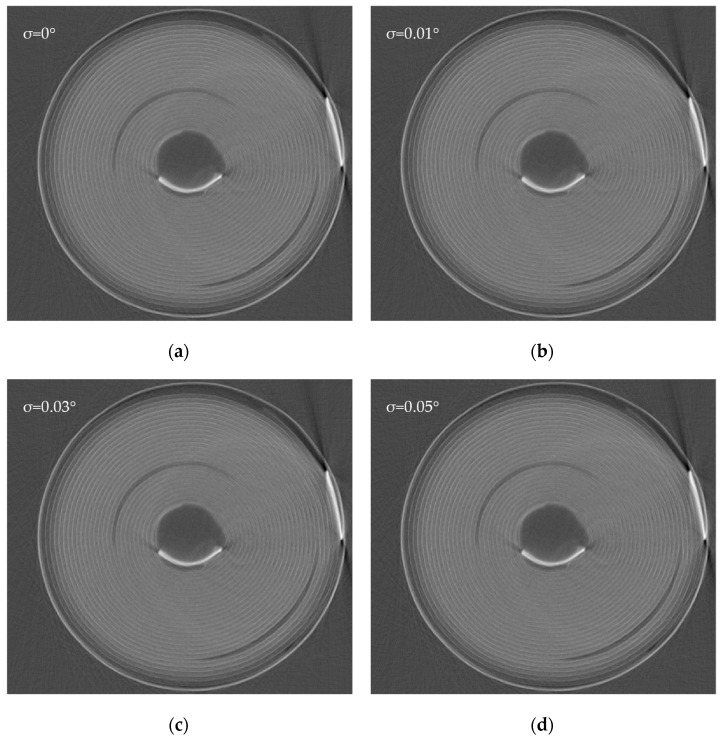

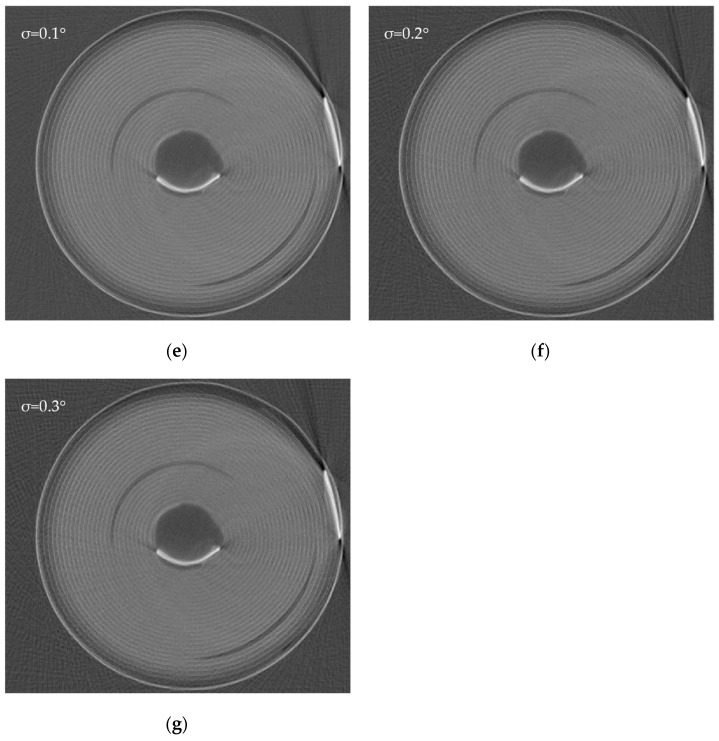
Images of the cross-sectional reconstruction of an 18650 lithium-ion battery under different angular errors. (**a**) Reconstruction result without added error. (**b**) Reconstruction result with added angular error, σ = 0.01°. (**c**) Reconstruction result with added angular error, σ = 0.03°. (**d**) Reconstruction result with added angular error, σ = 0.05°. (**e**) Reconstruction result with added angular error, σ = 0.1°. (**f**) Reconstruction result with added angular error, σ = 0.2°. (**g**) Reconstruction result with added angular error, σ = 0.3°.

Figure 8 displays the cross-sectional reconstruction images of lithium-ion batteries when the error takes different σ values. Similar to the change in *TV* and *AD* values, there are no visible changes in the images when the angle error σ is small. However, when the angle error σ exceeds 0.1°, the number of artifacts in the images significantly increases. In particular, many prominent striped artifacts are present in the image background. These artifacts can easily affect the observations of the internal structure of the battery, hence requiring attention. Therefore, when many irregular artifacts appear in the image background, it is important to note that the projection angle may have significantly deviated. When σ is 0.05°, even though no visible changes can be observed with the naked eye, the *TV* and *AD* values have increased by 1.5% and 0.6%, respectively, indicating that the image still has a certain amount of artifacts and distortion, which still requires attention.

A certain level of error in the rotation process of the rotary table is acceptable; however, in the actual operation of the rotary table, there is a small error in each rotation, and these errors add up over multiple rotations, leading to larger cumulative errors. Moreover, some random factors might cause a certain rotation to have a larger error, much higher than its precision level. These errors can be simply corrected by installing angle sensors to obtain good results, eliminating the need for complicated calculations later to improve the image quality. For cylindrical lithium-ion batteries at the cell layer level, according to the trends in the experimental *TV* value and the absolute difference, we should ideally control the rotation angle error within the range of σ less than 0.03°. According to the three sigma rule [42], the maximum error in rotary table rotation should be as low as possible, ideally below 0.09°.

## 5. Conclusions

The accuracy of projection angles is vitally important in XCT imaging, especially for XCT imaging of lithium-ion batteries with complex internal structures. This study adopts the FDK algorithm for cylindrical lithium-ion XCT cone-beam imaging tomographic reconstruction under different rotation errors using the *TV* and residual projection error as image evaluation indexes. In the experiments, we found that projection angle errors can lead to the appearance of a large number of strip-like artifacts in imaging; these artifacts are particularly noticeable in the background of the image and the *TV* can effectively reflect the number of artifacts in the image. Cumulative errors and random angle fluctuations may occur during the actual operation of the rotary table, potentially leading to projection angle inaccuracies. However, by installing angle sensors on the rotary table, we can effectively correct these errors. This eliminates the need for complex calculations to improve image quality later on. The background of the image can to some extent reflect whether the cone-beam CT projection angle is accurate. When there are many irregular stripe artifacts in the background of the fault reconstruction image, it is crucial to be aware that there might already be significant errors in the projection angles.

## Figures and Tables

**Table 1:** The *TV* and *AD* values at different σ values.

σ	0°	0.01°	0.03°	0.05°	0.1°	0.2°	0.3°
*TV* (×10^7^)	1.2701	1.2751	1.2765	1.2888	1.3112	1.3991	1.4175
*AD* (×10^10^)	1.3577	1.3583	1.3651	1.3658	1.4289	1.4528	1.5209

## Data Availability

The raw data supporting the conclusions of this article will be made available by the authors on request.
